# Coexistence of coinvading species with mutualism and competition

**DOI:** 10.1002/ecy.70039

**Published:** 2025-02-23

**Authors:** Naven Narayanan, Peter Lutz, Allison K. Shaw

**Affiliations:** ^1^ Simons Centre for the Study of Living Systems, National Centre for Biological Sciences Bangalore India; ^2^ Department of Ecology, Evolution, Behavior University of Minnesota Twin Cities Saint Paul Minnesota USA

**Keywords:** coexistence, coinvasion, competition, dispersal, integro‐difference equations, mutualism, mutualism dependence, range expansion

## Abstract

All interactions between multiple species invading together (coinvasion) must be accounted for to predict species coexistence patterns across space. Mutualisms, particularly, are known to influence species' population dynamics and their invasive ability (e.g., mycorrhizal fungi with partner plants). Yet, while modeling coinvasion, their role in mediating coexistence is overlooked. Here, we build a spatially explicit model of coinvasion of two competing plant species with a shared fungal mutualist to study how mutualism and competition interact to shape the local and regional coexistence of competitors. We observe four main results. First, mutualist presence generates regional coexistence between competitors even when local coexistence between them is impossible. Second, increasing partner mutualist dispersal leads to abrupt changes in competitor coexistence outcomes. Third, differences in mutualist partner dependence and competitive ability interact to produce a variety of local and regional coexistence outcomes. Fourth, asymmetry in the dispersal ability arising from dependence‐dispersal trade‐offs leads to greater exclusion of species less dependent on mutualist partners for growth. In toto, incorporating mutualism‐specific trait trade‐offs and dispersal asymmetries into coinvasion models offers new insights into regional coexistence and invasive species distributions.

## INTRODUCTION

Species invasions into novel habitats are increasing worldwide and have large‐scale ecological and economic impacts worldwide. Invasions can significantly modify biodiversity and ecosystem functioning by impacting local species richness and composition and modifying the biogeography of communities (Pyšek et al., 2012; Simberloff et al., 2013). While many studies have focused on single‐species invasions, far fewer have considered more realistic scenarios where multiple species invade together (termed co‐invasion) and how inter‐invader interactions shape community and ecosystem processes (Hejda et al., 2009). In particular, these interactions span the entire range of competitive (negative) to mutualistic (positive) (Jackson, 2015; Kuebbing & Nuñez, 2015). However, we lack a complete understanding of how invader‐invader interactions shape their coexistence across the habitat they invade and thereby their long‐term biogeography and spatial distributions.

Understanding the drivers of species coexistence, both local (at a single point in space) and regional (across the spatial ranges of the interacting species), is essential to understand the biogeography of communities and the distribution of the interacting species. Biotic drivers of local and spatial coexistence proposed include interactions with natural enemies or herbivores in the case of plants, life history, and competition–colonization trade‐offs among others (Amarasekare, 2003; Chesson, 1994; Fagan et al., 2005; Janzen, 1970; MacArthur, 1970; Tilman, 1982). Biotic interactions shaping regional coexistence, in particular, have often been limited to pairwise competition or predator–prey dynamics where competition between invading species leads to either local coexistence or species replacement; the predator behavior (generalism or specialism) can generate range overlaps or limits of the species (Case & Taper, 2000; Hochberg & Ives, 1999; Holt, 1984; Lutscher, 2019). However, more recently, the role of positive interactions has also been shown to shape the local coexistence between competing species (Bimler et al., 2018; Lanuza et al., 2018). These positive interactions, termed mutualisms, are ubiquitous ecological interactions between two or more species where the benefits exchanged lead to increases in their growth rates and thus their respective abundances (Bronstein, 2015; Vandermeer & Boucher, 1978).

Despite their ubiquity, the role of mutualisms in mediating the coexistence of competitors across spatial habitats (regional coexistence) is less well understood. This is a crucial gap in our knowledge as dispersal is a key trait shared by organisms across the tree of life (Clobert, 2012). When competing species disperse into novel habitats, the presence of mutualists can modify competition via third‐party interactions (Palmer et al., 2003). While classic non‐spatial competition theory tells us that species coexist or get excluded based on their competitive hierarchy, it cannot address whether species can coexist across habitats as “regional coexistence” is, by definition, a spatial phenomenon (Tilman, 1982). In the absence of a mutualistic partner, regional coexistence of competitors is only possible in spatially homogeneous environments via life history trade‐offs or competition‐colonization trade‐offs (Amarasekare, 2003; Tilman, 1994). However, spatial variation in mutualist densities generates heterogeneity contingent on the asymmetry in benefits it provides the competitors.

In mutualistic associations, species vary in the degree to which they require their partner's benefits to increase their own growth rates. This variation is termed as “mutualism dependence” and extends along a spectrum from complete dependence (obligacy) to independence from partner (Douglas & Smith, 1989; Janos, 2007; Ollerton, 2006). The degree of dependence on a mutualist partner can thus shape asymmetries in benefits received by competing species. Mutualism dependence often evolves to maximize the usage of partner benefits but trade‐offs can lead to trait loss resulting in reduced growth of a species when the partner is absent (Chomicki et al., 2020; Ellers et al., 2012; Siefert et al., 2019; Visser et al., 2010). Thus, for a given density of a mutualist partner, differences in mutualism dependence could lead to competitors obtaining different magnitudes of benefits from this shared mutualist partner. Differential benefits obtained by competitors from a shared mutualist have been shown to stabilize the local coexistence of competitors (Bever, 1999; Bever et al., 1997; Heijden et al., 2008; Umbanhowar & McCann, 2005).

In addition to influencing local coexistence by shaping competitor population growth, mutualisms also shape the spatial dynamics of interacting species (Fowler et al., 2023). Across a variety of taxa, positive interactions between two or more species lead to their repeated, successful invasions in a phenomenon termed “invasional meltdown” (Simberloff & Holle, 1999). This hypothesis has been borne out in several empirical examples including legume–rhizobial, plant–fungal mutualisms, and even plant–pollinator mutualisms (Lopez et al., 2021; Nuñez et al., 2009; Simberloff & Holle, 1999; Simonsen et al., 2017). For instance, the invasion and range expansion of pine species in parts of South America, New Zealand, and Hawaii are known to proceed only in the presence of their beneficial, mutualist ectomycorrhizal fungi (EM) (Dickie et al., 2017; Dickie & Reich, 2005; Hynson et al., 2013; Richardson et al., 2000; Traveset & Richardson, 2014). Indeed, increased dependence on their fungal partner is known to increase the invasive abilities of a tree species (Moyano et al., 2020, 2021). This dependence–invasion correlation can arise when more dependent species morphologically adapt to produce smaller seeds or lower seed wing loading (among other adaptations broadly termed Long Distance Dispersal [LDD] syndrome) that disperse further even at the cost of reduced competitive ability in low nutrient conditions (Allsopp & Stock, 1995; Correia et al., 2018; Greene & Johnson, 1993; Groom, 2010). These morphological adaptations leading to LDD crucially shape the distribution of plant species and colonization of new territories (Arjona et al., 2018). Indeed, it has been shown that mycorrhizal plants are more likely to have structures enabling LDD than non‐mycorrhizal plants (Correia et al., 2018; Vargas et al., 2012; Zhang et al., 2019). On one hand, LDD can increase the invasibility of a dependent species through increased dispersal but on the other hand could reduce its invasibility if it disperses much farther relative to its mutualist partner (Wenny, 2001). Thus, differential dispersal due to variation in LDD traits is one further mechanism that could shape the invasion and regional coexistence dynamics of competitors through priority and mutualistic effects (Fukami, 2015; Hess et al., 2019; Ploughe et al., 2020; Vannette & Fukami, 2014). Yet, we do not completely understand the interplay of competition, mutualism, and differential dispersal ability in shaping the invasion and the local or regional coexistence of coinvading species. Incorporating mutualistic interactions and mutualism‐associated LDD will paint a more realistic picture of the expected long‐term spatial distributions of these coinvading species as well as the conditions for their regional coexistence.

Here, we build a spatially explicit mathematical model of two coinvading competitors (e.g., congeneric plant species) with a shared mutualist partner (e.g., EM fungi or rhizobia) to understand, first, how the interplay of species interactions along with dispersal drive the conditions for locally and/or regional coexistence of competitors. Second, we consider scenarios where the shared mutualist disperses faster or slower than the competitors to identify how the relative dispersal abilities of the three interacting species drive the coexistence and spatial distribution of the coinvaders. Third, we implement empirically observed correlations between a species' mutualism dependence and dispersal ability and ask how they modulate these regional coexistence outcomes.

## METHODS

### Model

We build a system‐agnostic model of competition and mutualism, drawing inspiration from plant–fungal and plant–rhizobial models of mutualisms. We consider two very similar competing species (e.g., congeneric plant species) such that they have similar resource requirements, habitat requirements, and share the same mutualist partner species (rhizobia or EM fungi).

We model our system using a set of Integro‐Difference Equations (IDEs) that incorporate growth, species interactions, and dispersal of species. The growth and dispersal phases repeat in a cycle, over discrete time steps across continuous one‐dimensional space. Growth occurs from t to t+T, followed by dispersal at t+1, where t is a given year or generation. “*T*” is some period of time less than a year (or generation), that is, 0<T<1. Growth is modeled by coupled Ordinary Differential Equations (ODEs) for the three species while the dispersal of each species is governed by their own dispersal kernel (Narayanan & Shaw, 2024). We track the speed at which species expand into new regions in space along with how their population densities are spatially distributed.

In our model species, $F_{1}$ and $F_{2}$ are the focal plant species competitors and P is the partner mutualist of both $F_{1}$ and $F_{2}$. $F_{1}$ and $F_{2}$ differ both in their dependence on *P* (and thus receive different magnitudes of mutualistic benefits) and in competitive ability. Because we envision our model to mimic plant–microbial interactions, we assume that the degree of dependence would modify how well a mutualist partner is used for resources (e.g., nitrogen or phosphorus) which in turn enables them to be more competitive for a second, nonmutualism‐associated resource such as water or light.

**FIGURE 1:**
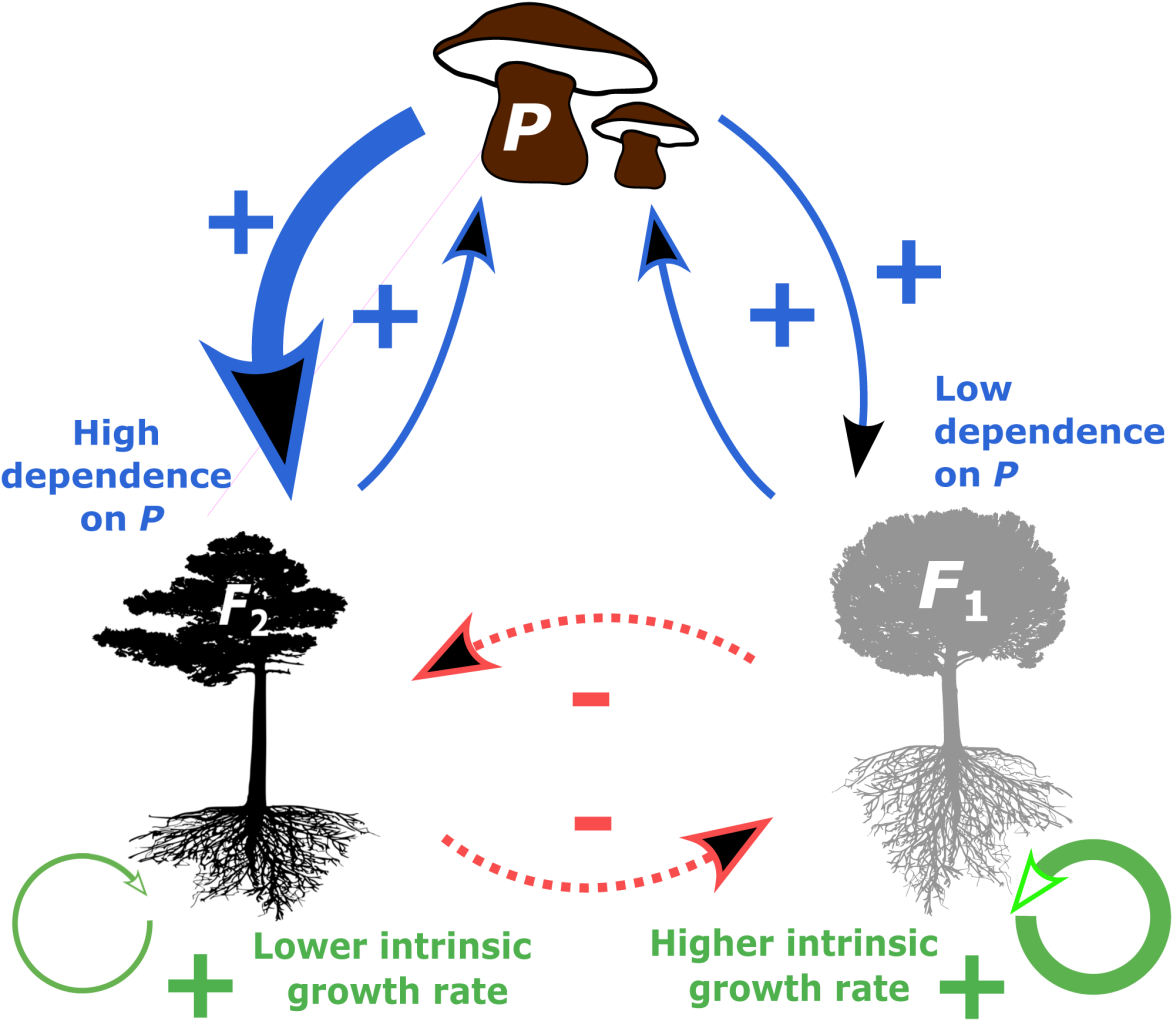
Schematic representation of interactions in the model. Species $F_{1}$ and $F_{2}$ are the focal competitor species (congeneric plant species) with shared mutualist *P* (fungal partner) where all species disperse and coinvade new territory. $F_{1}$ has greater dependence on mutualist partner *P* (in blue) but also lower intrinsic growth rate (in green) than $F_{2}$. Interspecific competition is denoted by dotted red arrows. Silhouette images contributed by T. Michael Keesey at phylopic.org and reused under Creative Commons license. The figure was made by author Naven Narayanan.

We assume, without loss of generality, that $F_{1}$ depends more on *P* than $F_{2}$ (see Figure 1). The general functional form of our IDEs is as follows:

(1a) $$\begin{array}{l} P_{t+1}\left(x\right)=\int_{-\infty }^{\infty }k_{P}\left(x-y\right)M_{P}\left(P_{t}\left(y\right),F_{1,t}\left(y\right),F_{2,t}\left(y\right)\right)\mathrm{dy}, \end{array}$$

(1b) $$\begin{array}{l} F_{1,t+1}\left(x\right)=\int_{-\infty }^{\infty }k_{F_{1}}\left(x-y\right)M_{F_{1}}\left(P_{t}\left(y\right),F_{1,t}\left(y\right),F_{2,t}\left(y\right)\right)\mathrm{dy}, \end{array}$$

(1c) $$\begin{array}{l} F_{2,t+1}\left(x\right)=\int_{-\infty }^{\infty }k_{F_{2}}\left(x-y\right)M_{F_{2}}\left(P_{t}\left(y\right),F_{1,t}\left(y\right),F_{2,t}\left(y\right)\right)\mathrm{dy}, \end{array}$$

where $k_{i}$ represents the dispersal kernel of species “*i*” (*i* = *P*, $F_{1}$, $F_{2}$), x and y are spatial coordinates after and before dispersal respectively, and $M_{i}$ is the nonlinear growth function describing the growth of species “*i*” at a point in space.

### Growth functions

We modeled the growth of the three species at a given point in the landscape using a set of ODEs. We modeled competition between $F_{1}$ and $F_{2}$ using classic Lotka‐Volterra dynamics with linear functional responses (Lotka, 1920). Mutualisms between *P* and $F_{1}$ (or $F_{2}$) are modeled as bidirectional consumer–resource interactions sensu (Holland & DeAngelis, 2009, 2010). The benefits obtained are modeled as a saturating function with partner density which captures physiological limits to uptake or handling of resources. Such saturation has been observed in fig‐fig wasp, ant‐treehopper, and ant‐aphid mutualisms (Addicott, 1981; Bronstein, 2001; Morales, 2000; Wright, 1989). In the absence of mutualists and competitors, a single species grows logistically to a carrying capacity. The equations are given as

(2a) $$\frac{\mathrm{dP}}{\mathrm{dt}}=P\left[r_{P}+\left(\frac{\alpha_{{\mathrm{PF}}_{1}}F_{1}}{h_{P}+F_{1}}+\frac{\alpha_{{\mathrm{PF}}_{2}}F_{2}}{h_{P}+F_{2}}\right)-d_{P}P\right],$$

(2b) $$\frac{{\mathrm{dF}}_{1}}{\mathrm{dt}}=F_{1}\left[\left(1-\delta_{F_{1}}\right)r_{F_{1}}+\delta_{F_{1}}\left(\frac{\alpha_{F_{1}P}P}{h_{F_{1}}+P}\right)-d_{F_{1}}F_{1}-\tau_{12}F_{2}\right],$$

(2c) $$\frac{{\mathrm{dF}}_{2}}{\mathrm{dt}}=F_{2}\left[\begin{array}{l} \left(1-\delta_{F_{2}}\right)r_{F_{2}}+\delta_{F_{2}}\left(\frac{\alpha_{F_{2}P}P}{h_{F_{2}}+P}\right)\\ -d_{F_{2}}F_{2}-\tau_{21}F_{1} \end{array}\right],$$

and are integrated from time “*t*” to “*t* + *T*” to obtain $M_{P},M_{F_{1}},M_{F_{2}}$ respectively. $\delta_{i}$ represents the mutualist partner dependence of species $F_{i}$. The total growth of a species (say $F_{1}$) is the sum of its own intrinsic growth and partner benefits weighted by its degree of dependence ($\delta_{F_{1}}$), resulting in some trade‐off between intrinsic growth versus benefit uptake from partner. Such trade‐offs between reproduction and root architecture have been observed in invasive forbs with different degrees of dependence on their mycorrhizal fungal partner (Seifert et al., 2009). $r_{i}$ is the intrinsic growth rates of species “*i*,” $\alpha_{\mathrm{ij}}$ is the maximal benefit received from species “*j*” by species “*i*,” $h_{i}$ is the half‐saturation constant of species “*i*,” *d*_*i*_s are the intra‐specific competition coefficients, and $\tau_{\mathrm{ij}}$ is the competitive effect on species “*i*” by species “*j*.” The magnitude of benefit received $F_{1}$ and $F_{2}$ is a combination of dependence (δ), α (maximum total benefit from partner), and partner density (*P*). In our model, we fix δ and α for both competitor species to describe facultative mutualisms which vary in dependence but not in α. Therefore, the variation in mutualistic outcomes comes from not just δ but the product of δ and the local density of the mutualist partner *P* (which in turn depends on its dispersal ability as well as benefits received from the competitor species). In our model, we do not explicitly model mutualism costs. We assume that benefits exchanged are “net benefits” and thus the costs of mutualism (i.e., resource acquisition of carbon, phosphorous, or nitrogen) are embedded in the expression itself.

### Dispersal kernel

Species dispersal is governed by their dispersal kernel, a probability density function describing the probability of an individual dispersing to and establishing at a location “*x*” given it started at another location “*y*.” More specifically, our kernel is a dispersal density kernel and in the 1D case, is equivalent to a dispersal distance kernel (Cousens et al., 2008; Nathan et al., 2012). We assumed that each species has a Gaussian dispersal kernel given as

(3a) $$k_{P}\left(x-y\right)=\frac{1}{\sqrt{2{\pi \sigma }_{P}^{2}}}e^{-\frac{{\left(x-y\right)}^{2}}{2\sigma_{P}^{2}}},$$

(3b) $$k_{F_{i}}\left(x-y\right)=\frac{1}{\sqrt{2{\pi \delta }_{F_{i}}\sigma_{i}^{2}}}e^{-\frac{{\left(x-y\right)}^{2}}{2\delta_{F_{i}}\sigma_{i}^{2}}},$$

where $\sigma_{P}^{2}$ is the variance of *P* and $\sigma_{F_{i}}^{2}$s are the variances of the dispersal kernel of the competitors. Gaussian dispersal kernels are a commonly used distriution to model dispersal (Bullock et al., 2017; Clark et al., 2003). While implementing dependence‐dispersal trade‐offs in our simulations, the kernels of the competitors are asymmetric. When these trade‐offs are not implemented, we ignore their $\delta_{F_{i}}$ values and set them to 1 thus making the kernels symmetric. In our model, lower (or higher) dispersal ability corresponds to a narrower (or wider) probability distribution function, that is, a narrower (or wider) dispersal kernel. Asymmetries in species' dispersal kernels were described by differences in the variance of their kernels.

### Simulations

To simulate the spatial dynamics of the model, we initialized a one‐dimensional landscape. Mathematically, this model is defined for continuous space but is discretised for numerical simulations. We expect our results would translate to a simple 2D space version of the model, given an isotropic dispersal kernel and single point introduction of the species in space. All simulations begin with very low population densities for each species ($P=F_{1}=F_{2}=0.1$) at the center of the landscape with symmetric coinvasion in both directions. The initial densities are low for the parameter values chosen (say, e.g., *P*: $r_{P}=0.3$ and $d_{P}=0.1$ leads to a single species carrying capacity of 3 as it reduces to a logistic model). The initial density therefore is 30 times smaller than the expected final density of species. Each species' growth phase occurred for an arbitrary *T* time steps; changing this number does not qualitatively affect results. Dispersal then occurs thus completing a single iteration of the simulation. We ran each simulation for 500 iterations by which time steady state was reached, which we defined as when all three species reached a constant range expansion speed at the edge and the population density of species behind their range edge reached equilibrium size. In our model, there are no interactions of spreading populations with the edge of the predefined habitat space. We defined a species' range as the total size of habitat occupied by the spreading species at the equilibrium at the end of the simulation. We then determined range expansion speed by subtracting the range edge location for the previous iteration from the one for the current iteration. We also defined a coefficient for spatial dominance of a species ρ which allowed us to determine what type of coexistence arose between the competitors. We define ρ as $R_{F_{1}}-R_{F_{2}}/R_{F_{1}}\cup R_{F_{2}}$, where $R_{F_{1}}$ and $R_{F_{2}}$ are the ranges of $F_{1}$ and $F_{2}$, respectively. The numerator describes the difference in the range size between the two species. Values greater than 0 indicate that *F*_1_'s range is larger than $F_{2}$ while the reverse is true when ρ<0. The denominator depicts the total size of space where either $F_{1}$ or $F_{2}$ (or both) is (are) present. Based on ρ's value, we can identify what sort of coexistence outcome can be expected between the competitors (see Appendix S1: Table S1 for expected ranges of ρ for different coexistence outcomes). Table 1 includes the parameter values used in all simulations. All simulations were performed in Matlab Version 2019a. All data and code located in the Zenodo repository (Narayanan et al., 2023).

**TABLE 1:** Model parameters and values used in simulations.

Symbol	Description	Simulation values
P	Population density of species P	…
$F_{1}$	Population density of species $F_{1}$	…
$F_{2}$	Population density of species $F_{2}$	…
$\tau_{\mathrm{ij}}$	Competition coefficient of species $F_{j}$ on $F_{i}$ (i,j=1,2;i≠j)	$0\leq \tau_{\mathrm{ij}}\leq 0.4$
$\delta_{F_{1}}$	Dependence on mutualism for growth of species $F_{1}$	0.9
$\delta_{F_{2}}$	Dependence on mutualism for growth of species $F_{2}$	0.1
$r_{i}$	Intrinsic growth rate of species i ($i=P,F_{1},F_{2}$)	0.3
$\alpha_{\mathrm{iP}}$	Mutualist benefits provided by species *P* to $F_{i}$ ($i=F_{1},\text{or}\;F_{2}$)	0.5
$\alpha_{\mathrm{Pj}}$	Mutualist benefits provided by species $F_{i}$ to *P* ($j=F_{1},\text{or}\;F_{2}$)	0.01
$h_{i}$	Half saturation constant of benefits provided to species i ($i=P,F_{1},F_{2}$)	0.3
$d_{i}$	Coefficient of intraspecific competition i ($i=P,F_{1},F_{2}$)	0.1
$\sigma_{i}^{2}$	Variance of dispersal kernel for species *i* (*i* = $F_{1},F_{2}$)	0.05
$\sigma_{P}^{2}$	Variance of dispersal kernel for species *P*	Variable (0–0.1)

### Scenarios

We ran four sets of simulations:

*Coexistence outcomes with symmetric dispersal kernels (Set 1)*: We compare the coexistence outcomes of competitors in cases where a mutualist coinvades with them versus is absent from the landscape. Here we assume that the dispersal kernels of all three species are equal, that is, symmetric kernels.

*Coexistence outcomes with different partner dispersal abilities, that is*, $\sigma_{P}^{2}$ *(Set 2)*: To understand how increasing dispersal ability of *P* altered the coexistence of competitors under different cases of competition (or niche overlaps) (i.e., strong [high overlap], intermediate [medium overlap], and weak [low overlap] competition coefficients where both species were equivalently competitive [$\tau_{\mathrm{ij}}\approx \tau_{\mathrm{ji}}$]), we swept over $\sigma_{P}^{2}$ for a range of values from low to high dispersal ability (i.e., [0, 0.099] with step size 0.001). In other words, we assume here that each species reduced the growth rate of their competitor to a similar extent. We then calculated the coefficient of spatial dominance (ρ) between the competitors. This coefficient ρ can be understood as the dominance of a species in terms of its relative range size compared with its competitor scaled by the total joint range size of the two competitors. ρ allows us to determine what type of coexistence arose between the competitors and is defined mathematically as $R_{F_{1}}-R_{F_{2}}/R_{F_{1}}\cup R_{F_{2}}$ (For different coexistence types, see Outcomes).

*Coexistence outcomes for differential competitive abilities between* $F_{1}$ *and* $F_{2}$ *(Set 3)*: Next, for a given dispersal ability of *P* (i.e. $\sigma_{P}^{2}$ is constant), we ask how differences in competitive ability (i.e., values of $\tau_{\mathrm{ij}}$ and $\tau_{\mathrm{ji}}$) influence the regional coexistence outcomes of $F_{1}$ and $F_{2}$ when *P* spreads (1) slower than, (2) equal to, or (3) faster than the competitors ($F_{1}$ and $F_{2}$) which have equal dispersal abilities (i.e., $\sigma_{F_{1}}=\sigma_{F_{2}}$). Asymmetries in dispersal kernels between mutualist partners (i.e., *P* and $F_{1}$ or *P* and $F_{2}$) are often observed in tree‐fungal mutualisms where tree seeds are often wind‐dispersed ∼100−200 m whereas fungal spores typically disperse in the order of ∼1−10 m (Galante et al., 2011; Greene & Johnson, 1993; Peay et al., 2012). However, the dispersal abilities of $F_{1}$ and $F_{2}$ still remain equal ($\sigma_{F_{1}}=\sigma_{F_{2}}$).

*Coexistence outcomes in the presence of Dependence‐Dispersal trade‐offs (Set 4)*: Finally, we incorporate dependence‐dispersal trade‐offs where increased dependence on a mutualist corresponds to greater dispersal ability through investments in LDD apparatus (Correia et al., 2018). This generates asymmetry in the dispersal ability of the competitors. We then study the interplay of asymmetric competitor dispersal with mutualist's (*P*) dispersal ability in shaping the regional coexistence of the competitors. We fixed values of dependence $\left(\delta_{F_{1}},\delta_{F_{2}}\right)=\left(0.9,0.1\right)$ on *P*. The difference in dependence modified the dispersal kernels of the competitors; more dependent competitors disperse further (Equations 3a and 3b). Our results are qualitatively robust to changes in the values of dependence, which vary from 0 to 1, given that the dependence values of $F_{1}$ and $F_{2}$ are sufficiently dissimilar. In this set of simulations, the dispersal kernels of all three species were different from one another. We ran all simulations across a wide range of competitive abilities of $F_{1}$ and $F_{2}$ (see Table 1 for values chosen).

### Outcomes

**FIGURE 2:**
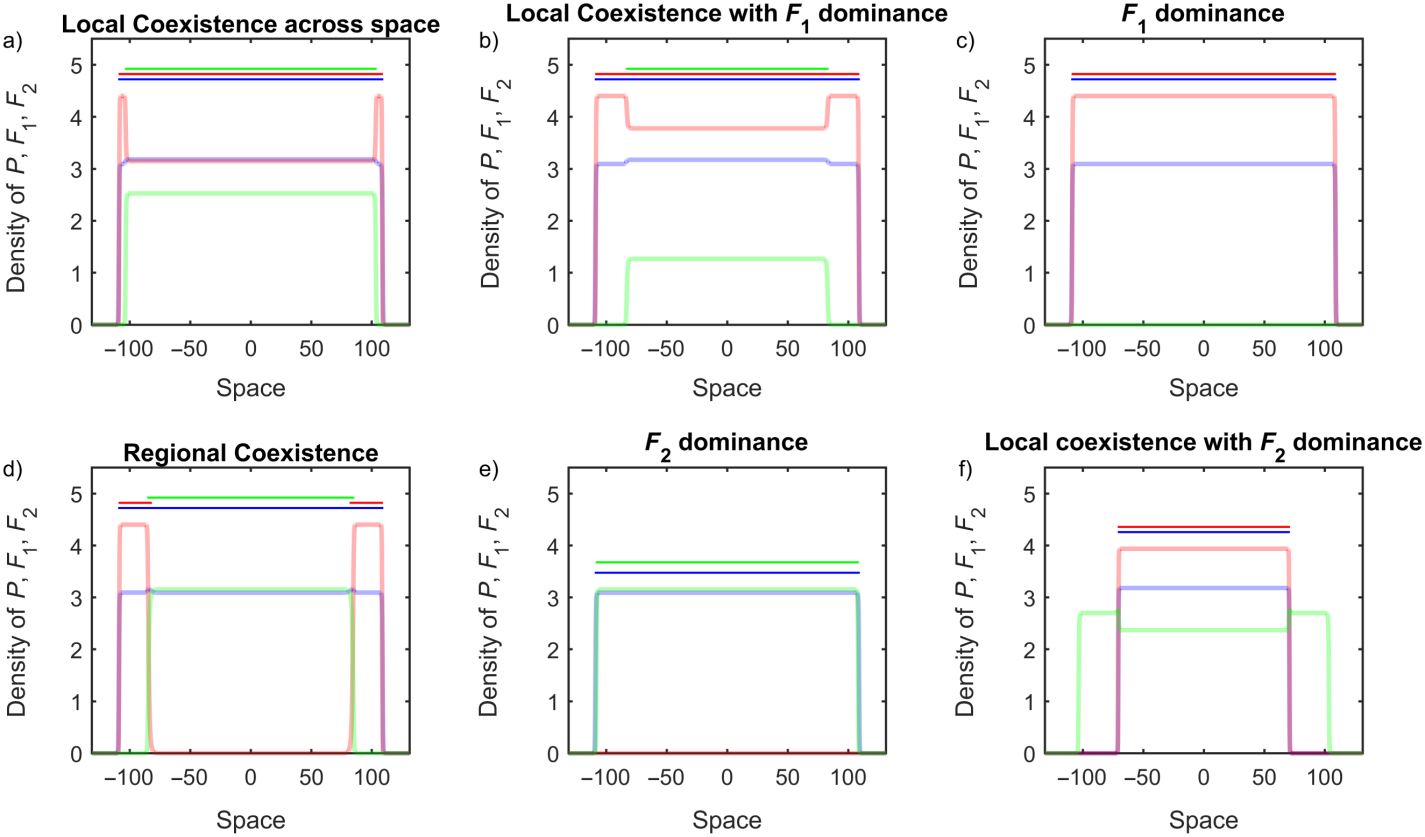
Set of qualitative outcomes observed. All figures are density versus space plots with species *P* in blue, species $F_{1}$ in orange and $F_{2}$ in green. (a) Local coexistence of competitors; (b) local coexistence with exclusion of $F_{2}$ by $F_{1}$ at their range edges; (c) competitive exclusion of $F_{2}$ across space; (d) regional coexistence of both species (but not local coexistence); (e) local coexistence with $F_{2}$ excluding $F_{1}$ at the edges; and (f) $F_{2}$ competitively excluding $F_{1}$ across all space. The horizontal lines above the graph indicate the ranges of each of the individual species (population at non‐zero densities). Parameter values used for these simulations are: $r_{i}=0.3\left(i=P,F_{1},F_{2}\right)$, $\delta_{F_{1}}=0.9,\delta_{F_{2}}=0.1$, $\sigma_{F_{1}}^{2}=\sigma_{F_{2}}^{2}=\sigma_{P}^{2}=0.05$ ($\sigma_{P}^{2}=0.02$ for subpanel f), $\left(\tau_{12},\tau_{21}\right)=\left(0.05,0.02\right),\left(0.05,0.05\right),\left(0.05,0.15\right),\left(0.15,0.05\right),\left(0.3,0.2\right),\left(0.02,0.02\right)$.

We classified the coexistence outcome of each simulation when a steady state was reached. If the population of $F_{2}$ was zero across all points in the landscape, we defined this as $F_{1}$ *dominance or win*, that is, competitive exclusion of $F_{2}$ and vice versa for $F_{1}$. When over 95% of the range of each species was also occupied by its competitor (i.e., co‐occurring), we defined it as *local coexistence*. In instances where $F_{1}$ (or $F_{2}$) excluded the other over a fraction (≥5%) of *F*_1_'s (or $F_{2}$) total range with local coexistence over the remaining range, we termed this as *local coexistence with* $F_{1}$ *or* $F_{2}$ *dominance*. This can be imagined as one species' range being a subset of the other's range. Finally, when we found $F_{1}$ and $F_{2}$ each exclusively occupying separate portions of the total occupied range due to either exclusion or differential dispersal abilities, we defined this as *regional coexistence* (see Figure 2 for illustrations of each coexistence outcome). All these different outcomes can be identified by calculating the value of ρ as defined earlier. Based on the range of values of ρ, we can identify what coexistence outcomes arises in each of our different scenarios (see Appendix S1: Table S1 for ρ values corresponding to outcomes).

## RESULTS

### Coinvasion of shared mutualist with competitors promotes their regional coexistence

**FIGURE 3:**
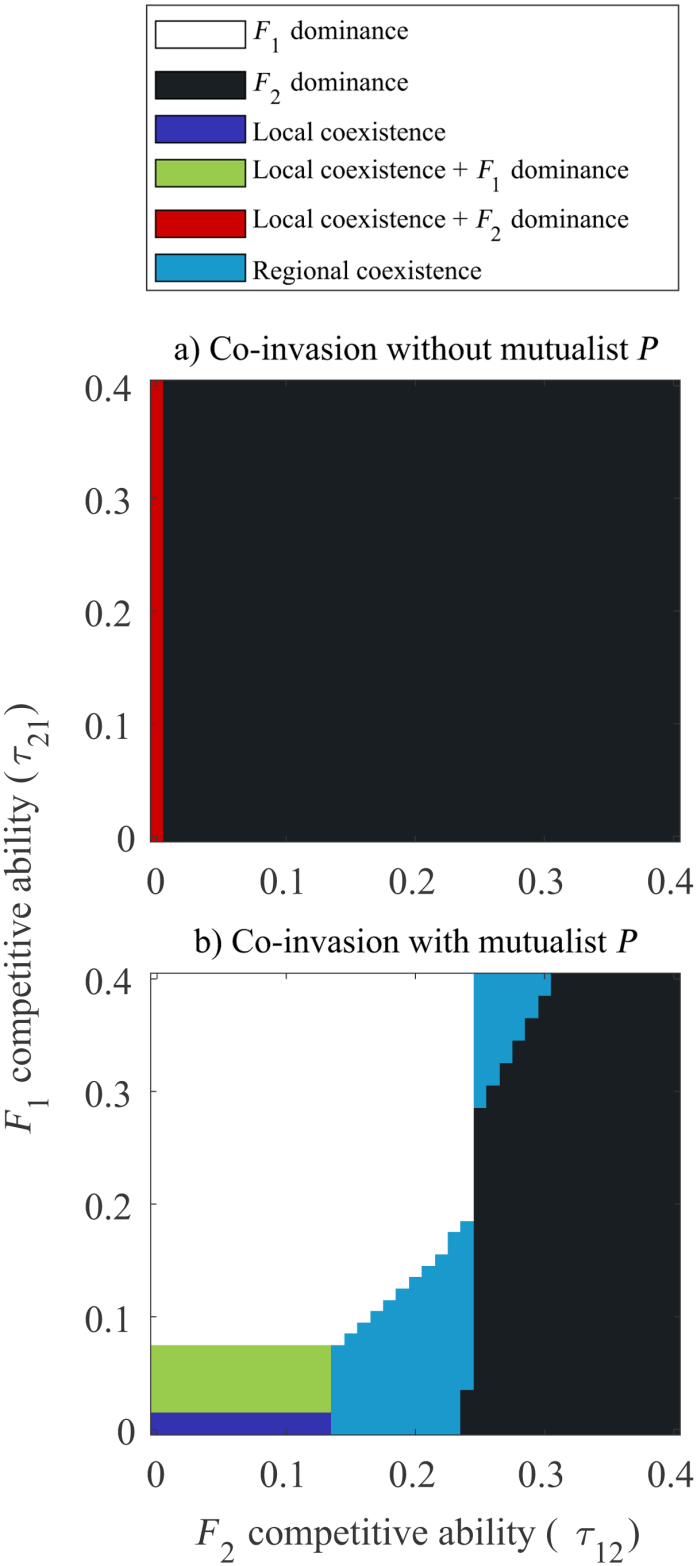
Coexistence of competitors of differing dependence arises in the presence of a coinvading mutualist. (a) Competitive exclusion of the more dependent $F_{1}$ without a mutualist; (b) different possible coexistence outcomes between the competitors in the presence of *P* for differing relative competitive abilities. Parameters: $r_{i}=0.3\left(i=P,F_{1},F_{2}\right)$, $\delta_{F_{1}}=0.9,\delta_{F_{2}}=0.1$, $\sigma_{F_{1}}^{2}=\sigma_{F_{2}}^{2}=\sigma_{P}^{2}=0.05$ (only for b).

We first ran simulations (Set 1) with coinvasion of $F_{1}$ and $F_{2}$ in the absence of *P*. We found that for all possible combinations of competition coefficients ($\tau_{12},\tau_{21}$) pairs, species $F_{2}$ always excluded $F_{1}$ across the landscape resulting in its complete extinction. Due to *F*_1_'s increased dependence of partner relative to $F_{2}$, its growth and competitive effect on $F_{2}$ was low, leading to its extinction. We then ran these coinvasion simulations in the presence of spreading mutualist (*P*). We found that *P*'s presence altered coexistence outcomes (Figure 3). When $\tau_{12}>>\tau_{21}$, we find similar to earlier that $F_{2}$ locally and spatially excludes $F_{1}$. On the other hand, when $\tau_{12}<<\tau_{21}$, $F_{1}$ wins and excludes $F_{2}$ across its range. Here, $F_{1}$ receives more benefits from *P* that $F_{2}$ and this, combined with its better competitive ability excludes $F_{2}$. When both species are weak competitors $\tau_{12}\approx \tau_{21}\in \left[0,0.07\right]$, they locally coexist due to *P* providing unequal buffers to each competitor's growth. Finally, when $\tau_{12}≳\tau_{21}$, we observe regional coexistence where $F_{2}$ always outcompetes $F_{1}$ in regions of common occurrence but $F_{1}$ spreads faster across space thereby partially escaping competition from $F_{2}$. *P* provides benefits which increases *F*_1_'s growth which in turn increases its spread speed and greater dispersal across space (Lutscher, 2019; Narayanan & Shaw, 2024). Thus, mutualisms promote both local and regional coexistence between competitors in scenarios where exclusion might have occurred in their absence.

### Increasing mutualist dispersal leads to qualitatively different coexistence outcomes in different competitive environments

**FIGURE 4:**
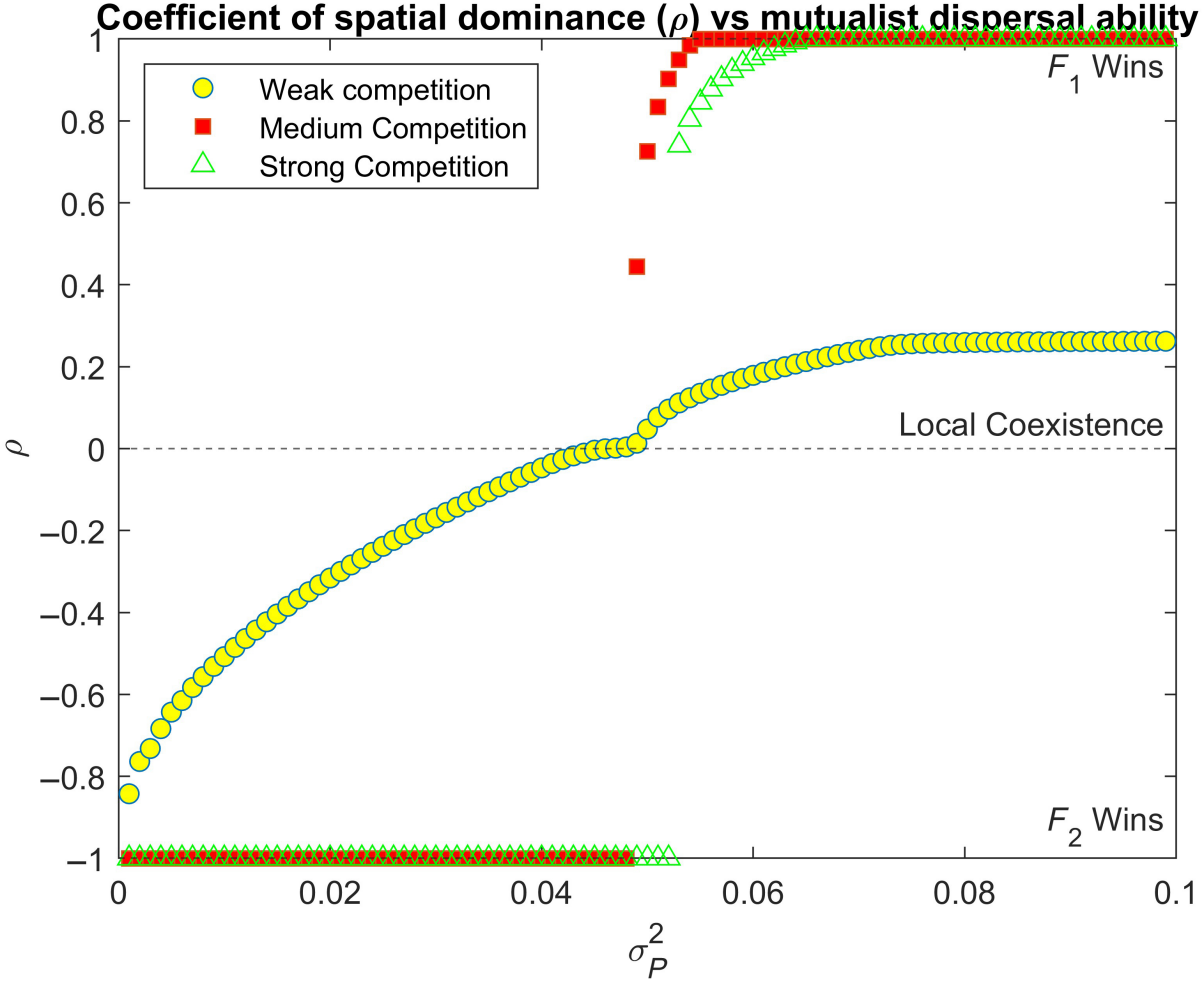
Increasing mutualist dispersal ability alters coexistence type in qualitatively different manners based on competition coefficients. When competition coefficients are low (yellow circles), regional coexistence outcome (denoted by ρ) shifts from $F_{1}$ exclusion to local coexistence with $F_{1}$ dominance. When the competition coefficients are intermediate (red squares) or strong (open green triangles), abrupt shifts arise from $F_{1}$ exclusion to $F_{2}$ exclusion with regions of regional coexistence for small regions of $\sigma_{P}^{2}$. Parameters chosen: $r_{i}=0.3\left(i=P,F_{1},F_{2}\right)$, $\delta_{F_{1}}=0.9,\delta_{F_{2}}=0.1$, $\sigma_{F_{1}}^{2}=\sigma_{F_{2}}^{2}=0.05$ ($\tau_{12},\tau_{21}=\left(0.02,0.02\right),\left(0.2,0.15\right),\left(0.37,0.29\right)$) for low, intermediate, and high competition respectively.

Next, we relaxed the assumption that all species had the same dispersal kernel and ran simulations to identify how mutualist dispersal ability could shape regional coexistence outcomes. We change the dispersal kernel of *P* relative to $F_{1}$ and $F_{2}$ while holding them constant (Set 2). Differences in dispersal ability between mutualistic partners of different taxonomic groups (e.g., plant and fungi) have been well documented and can vary to multiple orders of magnitude (Galante et al., 2011; Peay et al., 2012). We modified the variance of *P*'s dispersal kernel ($\sigma_{P}^{2}$) from 0.001 (relatively low dispersal relative to competitors) to 0.099 (relatively high dispersal ability) and studied its effect on regional coexistence of the competitors by measuring the spatial dominance of the competitors ρ. We found that when competition between both species was intermediate or high, increasing mutualist dispersal resulted in sharp transitions between outcomes from exclusion of more dependent species ($F_{1}$) to less dependent species ($F_{2}$) with a narrow region of intermediate values of $\sigma_{P}^{2}$ resulting in regional coexistence (Figure 4).

On the other hand, when $F_{1}$ and $F_{2}$ were in weak competition, there was a slight increase in ρ with increasing *P*'s dispersal ability (increasing $\sigma_{P}^{2}$), but neither competitor excluded the other. Rather, increasing $\sigma_{P}^{2}$ simply altered which species dominated at the edge of the coinvading community's range but maintained local coexistence at the core of their ranges.

### Differences in competitive ability result in varied coexistence outcomes of the competitors with equal dispersal kernels

**FIGURE 5:**
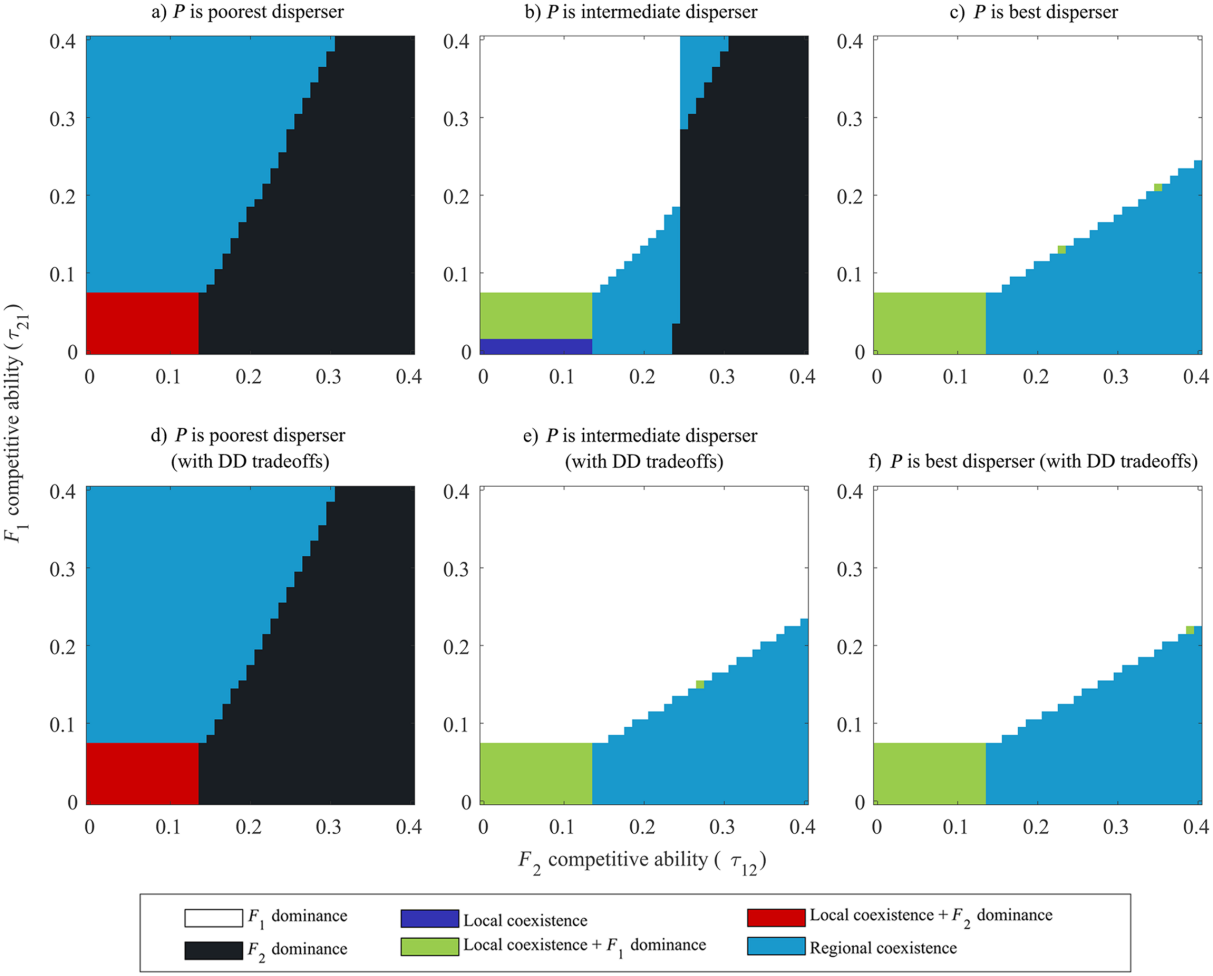
Coexistence outcomes are shaped by mutualist's dispersal ability and asymmetry in competitors' dispersal kernels arising from dependence‐dispersal trade‐offs. In (a–c), we assume $F_{1}$ and $F_{2}$ have symmetric dispersal kernels ($\sigma_{F_{1}}^{2}=\sigma_{F_{2}}^{2}$) and in (d–f), we assume $F_{1}$ and $F_{2}$ have asymmetric dispersal kernels ($\sigma_{F_{1}}^{2}\neq \sigma_{F_{2}}^{2}$). We consider *P* to have lower ($\sigma_{P}^{2}<\sigma_{F_{1}}^{2},\sigma_{F_{2}}^{2}$), similar ($\sigma_{P}^{2}\approx \sigma_{F_{1}}^{2},\sigma_{F_{2}}^{2}$), and greater ($\sigma_{P}^{2}>\sigma_{F_{1}}^{2},\sigma_{F_{2}}^{2}$) dispersal ability than the competitors. When competitors are asymmetric dispersers, only two outcomes are observed. Parameters: $r_{i}=0.3\left(i=P,F_{1},F_{2}\right)$, $\delta_{F_{1}}=0.9,\delta_{F_{2}}=0.1$. $\sigma_{P}^{2}=0.01\left(a\right)0.05\left(b\right)0.075\left(c\right)$; (d–f)$\sigma_{F_{1}}^{2}=0.03,\sigma_{F_{2}}^{2}=0.02;\sigma_{P}^{2}=0.01\left(d\right),0.025\left(e\right),0.075\left(f\right)$.

In our previous result, we found that regional coexistence was influenced by *P*'s dispersal ability (i.e., magnitude of $\sigma_{P}^{2}$) as well as overall strength of competition between $F_{1}$ and $F_{2}$. Here, we relaxed the assumption that $F_{1}$ and $F_{2}$ were equivalent competitors and explored how differences in competitive ability between $F_{1}$ and $F_{2}$ could influence coexistence outcomes (Figure 5a–c). We chose to study these outcomes for three different values of dispersal for *P* (i.e., $\sigma_{P}^{2}=0.01,0.05,0.075$) (Set 3).

We found that results greatly varied based on $F_{1}$ and $F_{2}$'s relative competitive ability. When *P*'s dispersal was slower than the competitors ($\sigma_{P}^{2}<\sigma_{F_{1}}^{2}=\sigma_{F_{2}}^{2}$), there were three possible outcomes. When $\tau_{12}>\tau_{21}$, $F_{2}$ excluded $F_{1}$ across space due to its lower dependence on *P* for growth and its relatively equal (if not greater) competitive ability to $F_{1}$. When competition was weak, that is, $\tau_{12}\approx \tau_{21}\in \left[0,0.07\right]$, $F_{2}$ locally coexisted with $F_{1}$ yet excluded it at $F_{2}$s range edge. However, when $\tau_{12}<\tau_{21}$, we find that both species regionally coexist. In these simulations, $F_{2}$ spreads faster than $F_{1}$ due to lesser dependence on the slow spreading *P* but get excluded behind their range front by $F_{1}$. Here, $F_{2}$ is not a strong competitor and $F_{1}$ growth is buffered by *P*.

When *P*'s dispersal is equal to the competitors ($\sigma_{P}^{2}=\sigma_{F_{1}}^{2}=\sigma_{F_{2}}^{2}$), we see a shift in coexistence. While we still see $F_{2}$ excluding $F_{1}$ when $\tau_{12}>>\tau_{21}$, we also see regions where $F_{1}$ excludes $F_{2}$ when $\tau_{12}<<\tau_{21}$. When competition is very low, both species locally coexist across their ranges while for slightly higher values of $\tau_{21}$, local coexistence with $F_{1}$ dominance at its range edge is observed.

Interestingly, the zone of regional coexistence occupies two separate, unconnected regions in the ($\tau_{21},\tau_{21}$) space. When both species are poor competitors ($\tau_{21},\tau_{21}<0.25$), regional coexistence arises when $\tau_{12}≳\tau_{21}$. In such cases, $F_{2}$ is the stronger competitor and hence outcompetes $F_{1}$ at the center of their ranges. However, as competition is weak, both competitors are at a high enough density for *P* to receive greater benefits. This is turn leads to large benefits provided to $F_{1}$ resulting in its faster spread thereby occupying the edges of co‐invaders' ranges. On the other hand, when competition is strong (and $\tau_{12}≲\tau_{21}$), regional coexistence is observed but with $F_{1}$ dominating at the centre of the range and $F_{2}$ existing on range edges. This is because low densities of $F_{1}$ and $F_{2}$ lead to reduced densities of (and benefits supplied to) *P*. This results in lower benefits provided to the more dependent $F_{1}$. Hence, $F_{1}$ spreads slower but still manages to regionally coexist with $F_{2}$ due to its superior competitive ability. Thus, mutualism influences population dynamics during the process of invasion leading to qualitatively different forms of regional coexistence.

When *P*'s dispersal is greater than the competitors ($\sigma_{P}^{2}>\sigma_{F_{1}}^{2}=\sigma_{F_{2}}^{2}$), there is another abrupt shift in the coexistence outcomes. When $\tau_{12}<\tau_{21}$, as opposed to regional coexistence (seen above), we find $F_{1}$ excluding $F_{2}$. Although we find cases where local and regional coexistence arise, we do not find scenarios where $F_{2}$ excludes $F_{1}$.

### Dispersal‐dependence trade‐offs increase the zone of regional coexistence of the competitors

Finally, we incorporated asymmetries in the dispersal kernels of the competitors that arise from adaptations correlated with the degree of dependence on *P* and performed simulations for different dispersal abilities of *P* (Set 4). Here too, we see an abrupt transition in coexistence outcomes based on *P*'s dispersal ability (Figure 5d–f). When *P*'s dispersal was slower than the competitors ($\sigma_{P}^{2}<\sigma_{F_{2}}^{2}<\sigma_{F_{1}}^{2}$), we see either local coexistence with $F_{2}$ dominance, $F_{1}$ exclusion, or regional coexistence similar to the case when the competitors' dispersal kernels were similar (Figure 5a). However, when *P*'s dispersal ability was intermediate ($\sigma_{F_{1}}^{2}>\sigma_{P}^{2}>\sigma_{F_{2}}^{2}$), there was a shift in coexistence outcomes where $F_{1}$ excludes $F_{2}$ when $\tau_{12}<\tau_{21}$ but the competitors regionally coexist when $F_{2}$ is the better competitor with local coexistence (with $F_{1}$ dominance) occurring when both $\tau_{12}$ and $\tau_{21}$ were low. This pattern was recapitulated even as we increased *P*'s dispersal ability, that is, $\sigma_{P}^{2}>\sigma_{F_{1}}^{2}>\sigma_{F_{2}}^{2}$. Thus, we find that differential competitor dispersal creates a sharper transition between the coexistence patterns that are generated with increasing $\sigma_{P}^{2}$.

## DISCUSSION

In this paper, we sought to understand how mutualistic interactions shaped the local and regional coexistence patterns of two coinvading competitor species. First, we found that the presence of a common mutualist dramatically alters the coexistence outcome between competing coinvaders, a result that matches previous observations while providing further insights. Variation in mutualism traits, that is, dependence (δ) engendered both local coexistence and thus co‐occurence (by buffering against competitive exclusion of weaker species at a location in space) as well as regional coexistence (by providing benefits which in turn increase growth and range expansion speeds of the more dependent species). Our study thus recapitulated previous results that elucidate the role of mutualism in mediating competition between competitors through population dynamic effects (Siefert et al., 2019; Umbanhowar & McCann, 2005). However, unlike previous studies, the spatially explicit design of our model provided us the additional insight that regional coexistence between unequal competitors was still possible during co‐invasion even if local coexistence was not (Figure 3). Patterns of co‐occurence and segregation between *Carduus nutans* L. and *C. acanthoides* L., two economically important invasive weeds in the United States, are known to arise from competitive interactions between both species (Allen & Shea, 2006; Rauschert et al., 2012). Further, this competition is mediated by mutualist pollinator species (Yang et al., 2011). Such mediation of plant–plant competition by mycorrhizal fungi has also been observed (Hartnett & Wilson, 1999; Scheublin et al., 2007). Our model incorporated competition and mutualism to understand the interplay between these interactions. We also found that competition acts alongside species spread resulting in the constriction of the range size of the poorer competitor. In natural systems as well, such reduction in range and extinction of invasive species arises when other competitive invaders are present (Simberloff & Gibbons, 2004). As our model predicted (e.g., Figure 2c,e), the population of the weaker competitor crashes leading to replacement by the stronger competitor across its range (Jäger et al., 2009; Morrison et al., 2007). Competition is a common mechanism invoked to generate species range borders (Case & Taper, 2000; Fowler & Levin, 1984). However, unlike past models with only antagonistic interactions, co‐invasion with a mutualist led to regional coexistence with the weaker competitor if it was a more dependent species.

Second, we found that coexistence outcomes of the co‐invaders is strongly impacted by the dispersal ability and distribution of the shared mutualist partner. Lower relative dispersal ability (to co‐invaders) of the mutualist could lead to a “missed mutualist” scenario wherein the less dependent competitor excludes the more dependent one. However, with increasing dispersal ability, the three species system showed coexistence patterns likely to occur due to an “enhanced mutualism” of these co‐invaders (Moles et al., 2022; Yu et al., 2022). We find that invaders with lesser dependence on a mutualist are more succesful competitors when mutualist dispersal ability and range expansion speeds are low, that is, low $\sigma_{P}^{2}$ (Figure 4; left half wherein $\sigma_{P}^{2}<0.05$). These model predictions are in line with correlative empirical evidence showing that species that successfully invade habitats with disturbances and low mycorrhizal mutualist densities tend to have low mycorrhizal dependence (Bunn et al., 2015; Pendleton & Smith, 1983; Seifert et al., 2009). In our model, we found these results to be true in the case of intermediate or strong competitive environments. However, our model further predicts that increasing dispersal ability of the mutualist (*P*) results in a transition from the less dependent to the more dependent species outcompeting the other (Figure 4; right half wherein $\sigma_{P}^{2}>0.05$). Presence of the mutualists allows the more dependent co‐invader to derive greater benefits from the mutualist leading to increased growth and greater competitive ability. Past studies in several plant taxa have shown that in strong competitive environments and in the presence of a shared mutualist, species that derive more benefits from the mutualist have a competitive advantage (Hartnett & Wilson, 1999; Scheublin et al., 2007). This translates to the more dependent species ($F_{1}$) completely outcompeting the less dependent species ($F_{2}$) across the entire spatial range. Interestingly, our model predicted that when invader‐invader competition was weak ($\tau_{12},\tau_{21}<0.05$), increased dispersal of *P* did not change patterns of coexistence but altered simply which species dominated at the range edge (Figure 4). By devising system specific metrics of competition, future empirical studies could test whether regional coexistence is maintained in the presence or absence of a mutualist.

Third, competition outcomes are shaped both by relative competitive ability and the presence (or absence) of DD trade‐offs even for some fixed dispersal ability of the mutualist($\sigma_{P}^{2}$; Figure 5). Our model predicted that the set of coexistence (or exclusion) outcomes was more diverse when the dispersal ability of the mutualist is relatively equal to the competitors (Figure 5). Here, the mutualist is present at low densities (similar to the competitors) at their range front and all three species are in the transient phase of their growth. This transience allows for several equilibria to be accessed based on the species' relative competitive abilities and mutualist dependence. However, it is unlikely in nature that we find plant and fungal dispersal occurring over the same spatial scale. Plant seed dispersal is often orders of magnitude larger than fungi per dispersal event (Galante et al., 2011; Greene & Johnson, 1993). In cases where fungal species are newly established alongside the coinvading competitors, our model predicted range expansion and coexistence dynamics should proceed as shown in Figure 5a (or Figure 5d). Such examples of co‐invasion with fungi is observed in *Pinus* genus (Dickie et al., 2010). On the other hand, the fungal range could be larger than the co‐invaders if it is native to the habitat or if it has several dispersal events in the same time period as a single plant dispersal event. Alternatively, the presence of dormant fungal spores present in the range that forms associations with the co‐invaders could lead to coexistence scenarios such Figure 5c (Glassman et al., 2016; Hopkins & Bennett, 2023). Finally, we also found that dispersal‐dependence trade‐offs that led to larger dispersal kernels of more dependent species did not provide qualitatively novel coexistence outcomes (Figure 5d–f). Rather, there was a sharper transition from one set of coexistence outcomes to the other (Figure 5d–e) with regional coexistence being the predicted outcome for a wide range of values of relative competitive ability (i.e., $\left(\tau_{12},\tau_{21}\right)$) unlike the scenario with no DD trade‐offs (Figure 5b). Without DD trade‐offs, $F_{2}$ could exclude $F_{1}$ when it was the superior competitor (right side of Figure 5b) but with trade‐offs, $F_{1}$ dispersed farther allowing it to escape from the trailing $F_{2}$ even if $F_{2}$ is the superior competitor (right side of Figure 5e). These trade‐offs thus allowed for greater partitioning of the invaded habitat leading to regional coexistence. Although not presented explictly as such, our model also predicted outcomes of invader–native plant competition in the presence of a common mutualist. Such competitive interactions are observed globally with outcomes of these interactions potentially modifying future communities through soil legacy effects (Fahey & Flory, 2022; Putten et al., 2010; Reinhart & Callaway, 2006). In our model, scenarios where $\sigma_{P}^{2}>\sigma_{F_{1}}^{2}>\sigma_{F_{2}}^{2}$ are equivalent to $F_{2}$ invading a native plant–fungal mutualism and outcomes of local and regional coexistence should proceed similar results presented in Figure 5e.

Our results could also be interpreted in the context of mutualism trait evolution during invasion. By considering $F_{1}$ and $F_{1}$ as two variants (within a species) with different degrees of investment into mutualism traits, our results identified conditions during coinvasion in which mutualism should be selected for (i.e., $F_{1}$ dominance across space). On the one hand, selection could favor increasing dependence on mutualism during invasion if these interactions provide $F_{1}$ a fitness advantage over the less dependent ($F_{2}$) conspecific invading with it or being present in the native community (Rodrgíuez‐Echeverría et al., 2012; Rodríguez‐Echeverría et al., 2009). On the other hand, selection for lesser mutualism dependence could arise particularly in the presence of competitors, reduced mutualist availability in novel habitats or life history trade‐offs (Seifert et al., 2009; Shelby et al., 2016; terHorst et al., 2018). Indeed, evolution during invasion could lead to mutualism breakdowns between plant and symbiont and result in more exploitative symbiont phenotypes (Wendlandt et al., 2021). One could also more explicitly analyze the evolution of dispersal ability in models of invasion as opposed to preemptively assuming trade‐offs between dispersal ability and dependence (mutualism associated trait). Such correlated trait evolution during spatial expansion has been observed in soapberry bug following recolonisation of habitats following local extinctions (Comerford et al., 2023).

Our model also provides suggestions to prevent invasional meltdowns, an important management priority. In cases of co‐invasions, there is strong evidence that the removal of one non‐native invader allows for other non‐natives to invade or increase in abundance (Ballari et al., 2016; Pearson et al., 2016). For instance, removing just $F_{1}$ (or $F_{2}$) removes competitive pressure on the other species leading to increased invasion of the other species. On the other hand, removing both will lead to the slowdown of even *P* as its partner mutualists are absent. These suggestions are easy to make in our model as the interactions between the coinvading species are well known. In cases where all interactions are unknown, managers should look to manage more than one species to prevent any sort of unexpected secondary invasions (Pearson et al., 2016). If there are economic constraints on managing more than one species, our model suggests prioritizing detection and management of either species $F_{1}$ or $F_{2}$ in a more context‐dependent fashion where context here refers to *P*'s presence/absence in regions where $F_{1}$ and $F_{2}$ will invade. In cases where *P*'s range and/or dispersal ability is larger than either competitor, managing $F_{1}$ (i.e., the more dependent species) is more important to prevent invasional meltdown. However, when *P* is a slow spreader, controlling the spread of the less dependent species, that is, $F_{2}$ is more critical to manage invasions.

There are several possible extensions to our model one of which is relaxing the assumption that the landscape across which species spread is homogeneous. This implies our model does not generate coexistence patterns that arise due to spatial heterogeneity such as spatial storage effects (Amarasekare, 2003; Chesson, 2000). Abiotic variation across space is also an important generator of dispersing species' range limits. In this paper, we have motivated our model using plant–microbial interactions, but we believe IDEs can be used as a general framework to model mutualisms with other mechanisms (i.e., nutritional, pollination, defensive, or even dispersive). In nutritional, pollination, and defensive mutualisms, the benefit ultimately arises either due to increased growth, germination, and pollination rates or reduced mortality rates (in defensive mutualisms) whose effects can be described using difference or differential equations. Indeed, even in nutritional mutualisms, some studies propose a quadratic response of benefits that increase with partner density before decreasing and ultimately resulting in mutualism breakdowns (Gange & Ayres, 1999; Vannette & Hunter, 2011). For dispersive mutualisms, one could imagine the shape of the dispersal kernel (e.g., the variance) of a species change as a function of its partner disperser density. For instance, fruits whose seeds are dispersed by a frugivore will ultimately have a dispersal kernel tightly coupled with the frugivore's local density and its dispersal kernel. Further, mutualisms can themselves be context dependent and vary across space in how much benefits are exchanged between species (Chamberlain et al., 2014; Cunning & Baker, 2014; Drew & King, 2022). We will extend our work to focus on the effects of spatial variation in mutualistic benefit exchange which can then shape coexistence outcomes of invasive species. More broadly, a limitation of this model is that species interactions are phenomenological and thus do not delve into system‐specific mechanisms that differ between different mutualisms. A more mechanistic approach could parse these differences. For example, this model could be modified such that the mutualist partner is a shared pollinator that mediates the competition between the two plant species during the process of invasion. Since our model is phenomenological, we trade‐off system specificity (plant–pollinator/plant–fungal interactions) for a more general understanding of competitive and mutualistic phenomena (as it pertains to influencing species growth rate negatively or positively) and their influence on species coexistence across space. Finally, extending the model to incorporate spatial heterogeneities that play out across two spatial dimensions of space could create qualitatively different results than those seen in our model.

In conclusion, we showed that extending models of co‐invasion to include multiple biotic interactions leads to an array of local and regional coexistence outcomes We hope the theoretical framework presented here fosters further research into the role of mutualism as an important biotic driver of local and regional coexistence of coinvading communities.

## AUTHOR CONTRIBUTIONS

Naven Narayanan and Allison K. Shaw conceived the study. Peter Lutz and Naven Narayanan conducted simulations and plotted results under the supervision of Allison K. Shaw. Naven Narayanan wrote the first draft with significant contributions from Peter Lutz. All authors contributed substantially to revisions.

## CONFLICT OF INTEREST STATEMENT

The authors declare no conflicts of interest.

## Supporting information


Appendix S1:

## Data Availability

Data and code (Narayanan et al., 2023) are available on Zenodo: https://doi.org/10.5281/zenodo.8357360.
