# Supplementary material for: Coexistence of coinvading species with mutualism and competition
Source: Ecology. 2025 Feb 23;106(2):e70039. doi: 10.1002/ecy.70039 (PMC11848128; doi:10.1002/ecy.70039)
Supplement: Supplementary file 1 — Appendix S1: [file ECY-106-e70039-s001.pdf]

Ecology

Appendix S1

Coexistence of coinvading communities with mutualism and competition

Naven Narayanan, Peter Lutz, Allison K. Shaw

Section S1 Table of (ρ) expressions for different outcomes

We defined a coefficient for spatial dominance of a species ρ which allowed us to determine what type of coexistence arose between the the competitors. We define ρ as $\frac{R_{F_1}-R_{F_2}}{R_{F_1}\cup R_{F_2}}$ where R_{F_1} and R_{F_2} are the ranges of the F_1 and F_2 respectively. The numerator describes the difference in the range size between the two species. Values greater than 0 indicate that F_1's range is larger than F_2 while the reverse is true when $\rho < 0$. The denominator depicts the total size of space where either F_1 or F_2 (or both) is (are) present. The denominator (A $\cup$ B) takes different values based on whether F1 and F2 locally coexist (LC + F1 or F2 dominance) or regionally coexist (RC). We use the fact that if there is RC, then $R_{F_1} \cup R_{F_2} = R_{F_1} + R_{F_2}$ and is therefore greater than each of the individual species ranges. On the other hand, when the outcome is LC+F1 or LC+F2 , $R_{F_1} \cup R_{F_2} = min(R_{F_1}, R_{F_2})$. By plugging these values in, we can get the analytical expressions seen in Table S1. This leads to different "expected" value expressions of ρ for the different coexistence outcomes (as seen in Table S1). We then compare the "observed" ρ (calculated at the end of a simulation run as opposed to using the analytical expressions) with the ρ we would "expect" for either coexistence outcome. This is useful in certain cases where ρ values are similar for different coexistence outcomes. ρ = 0, for instance, implies local coexistence across the spatial domain (LC). However, in the extremely rare case that $R_{F_1} = R_{F_2}$, regional coexistence (RC) could also give rise to ρ = 0. Therefore, in simulations where rho is observed to be 0, we compare what the expected rho should be in a given simulation if it were RC or LC (based on the expressions in Table S1). We then plot these expected values of rho as a result of the different coexistence outcomes along with the observed value from the simulations in order to discern which coexistence mechanism leads to rho equalling 0 in a simulation

Shown below are the expected expressions of ρ. These expressions can be evaluated for each simulation and compared with the observed ρ to identify what coexistence outcomes are observed. The magnitude and sign of ρ provides us with a quantitative estimate of what outcome we should expect at the end of a simulation.

Table S1: Coefficient of spatial dominance (ρ)

Coexistence outcome	Expression for ρ	Expected value of ρ
F_1 wins	$\frac{R_{F_1}}{R_{F_1}}$	ρ = 1
F_2 wins	$\frac{-R_{F_2}}{R_{F_2}}$	ρ = -1
Local coexistence with F_1 dominance	1 - $\frac{R_{F_2}}{R_{F_1}}$	$0 < \rho < 1$
Local coexistence with F_2 dominance	$\frac{R_{F_1}}{R_{F_2}} - 1$	$-1 < \rho < 0$
Local coexistence everywhere	0	ρ = 0
Regional coexistence	$\frac{R_{F_1}-R_{F_2}}{R_{F_1}+R_{F_2}}$	$-1 < \rho < 1$

Section S2 Differentiating between whether observed ρ suggests local or regional coexistence

The metric we define for the coefficient of spatial dominance of the two competitors while invading and expanding i.e. ρ helps identify which of the coexistence outcomes we observe in each simulation of a given parameter set. When ρ is positive, the range size of F_1 is greater and when it is negative, the reverse holds true. $\rho = 1$ implies that F_1 completely outcompetes F_1 and no manner of coexistence (local or regional) is possible. When $\rho = -1$, the reverse is true and F_2 completely dominates the landscape. However, when $-1 < \rho < 0$, it is less easy to determine whether the competitors are regionally coexisting (RC) or if they locally coexist with F_2 dominating at the edges (LC+F_2). Similarly, when $0 < \rho < 1$, we require to differentiate between local coexistence with F_1 dominance at the range edges (LC+F_1) and regional coexistence. To verify results presented in Figure 2 in the main document (and corresponding text in the results section), we calculate the expected ρ for each of our simulations (increasing dispersal ability of P i.e. σ_P^2) and the three different types of competition (weak, intermediate, and strong) using expressions from Table S1. We then plot the observed ρ to check which of the two outcomes it matches - either RC or LC+F_2 if $-1 < \rho < 0$ else RC or LC+F_1 if $0 < \rho < 1$. We find that when competition is weak, the observed ρ aligns with the expected ρ associated with LC+F_1 (or F_2)

dominance but for intermediate and strong competition, the observed ρ aligns with the expected ρ associated with RC. Figure S1 shows expected and observed ρs corresponding to Figure 2 (i.e. symmetric dispersal kernels) and S2 shows the same results when the kernels are asymmetric due to dependence-dispersal tradeoffs.

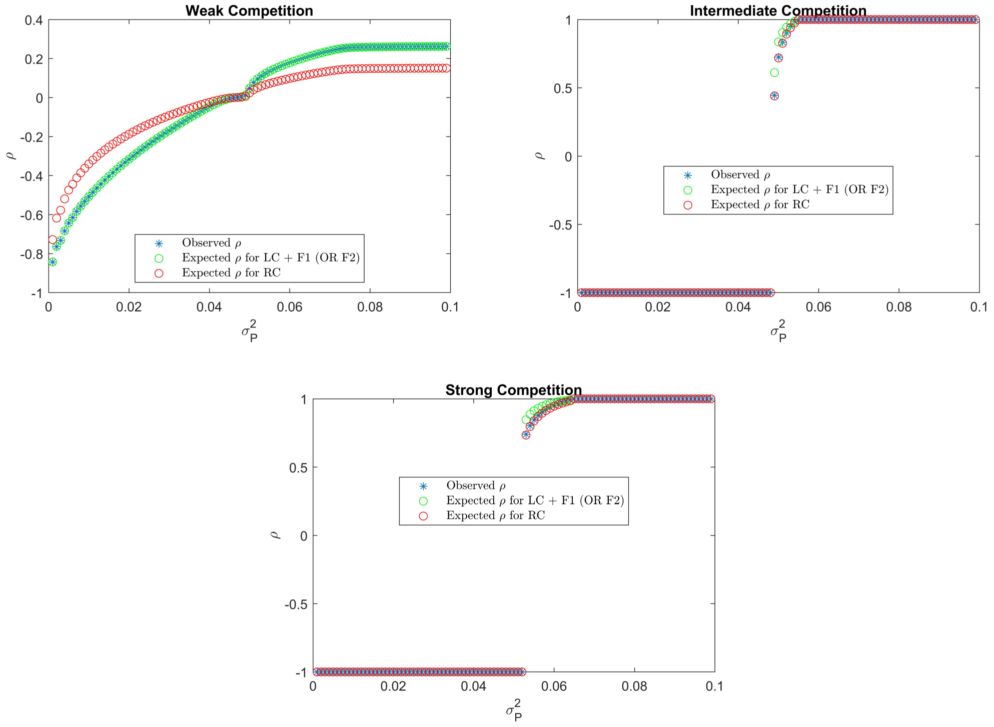

Figure S1: Correspondence of observed ρ in simulations to expected ρ of either local or regional coexistence when species kernels are symmetric. Subpanels a, b, and c show three different levels of competition (weak, intermediate, and strong). Open red circles indicate expected ρ for RC, open green circles are expected ρ for LC + F_1 (or F_2) and blue stars are observed ρ. Parameters chosen: $r_i = 0.3(i = P, F_1, F_2)$, $\delta_{F_1} = 0.9, \delta_{F_2} = 0.1$, $\sigma^2_{F_1} = \sigma^2_{F_2} = 0.05$ $(\tau_{12}, \tau_{21} = (0.02, 0.02), (0.2, 0.15), (0.37, 0.29))$ for weak, intermediate, and strong competition respectively.

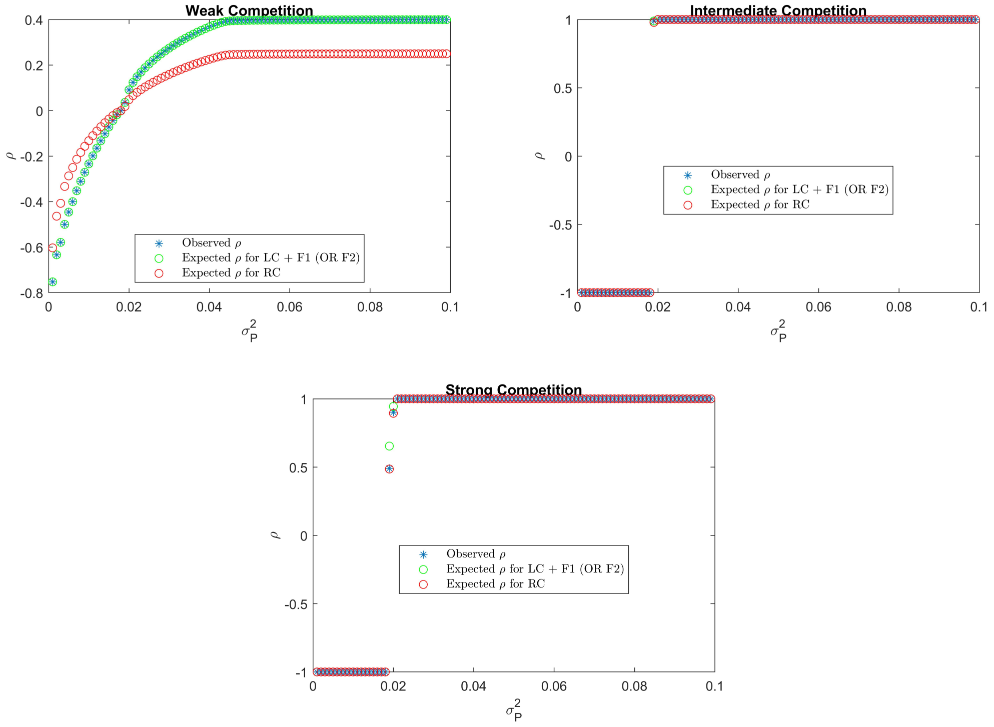

Figure S2: Correspondence of observed ρ in simulations to expected ρ of either local or regional coexistence when species kernels are asymmetric. Subpanels a, b, and c show three different levels of competition (weak, intermediate, and strong). Open red circles indicate expected ρ for RC, open green circles are expected ρ for LC + F_1 (or F_2) and blue stars are observed ρ. Parameters chosen: $r_i = 0.3(i = P, F_1, F_2)$, $\delta_{F_1} = 0.9, \delta_{F_2} = 0.1$, $\sigma^2_{F_1} = 0.03, \sigma^2_{F_2} = 0.02$ $(\tau_{12}, \tau_{21} = (0.02, 0.02), (0.2, 0.15), (0.37, 0.29))$ for weak, intermediate, and strong competition respectively.
